# RalBP1 and p19-VHL play an oncogenic role, and p30-VHL plays a tumor suppressor role during the blebbishield emergency program

**DOI:** 10.1038/cddiscovery.2017.23

**Published:** 2017-05-29

**Authors:** Goodwin G Jinesh, Ashish M Kamat

**Affiliations:** 1 Department of Urology, The University of Texas MD Anderson Cancer Center, Houston, TX 77030, USA

## Abstract

Cancer stem cells evade apoptotic death by blebbishield emergency program, which constructs blebbishields from apoptotic bodies and drives cellular transformation. Von Hippel–Lindau (VHL) plays both tumor suppressor and oncogenic roles, and the reason behind is poorly understood. Here we demonstrate that dimers and trimers of p19-VHL interact with RalBP1 to construct blebbishields. Expression of RalBP1, p19-VHL, and high-molecular weight VHL is required to evade apoptosis by blebbishield-mediated transformation. In contrast, p30-VHL plays a tumor suppressor role by inhibiting blebbishield-mediated transformation. Furthermore, target genes of VHL that suppress oxidative stress were elevated during blebbishield-mediated cellular transformation. Thus, RalBP1 and p19-VHL play an oncogenic role, whereas p30-VHL plays a tumor suppressor role during the blebbishield emergency program by regulating oxidative stress management genes.

## Introduction

The blebbishield emergency program helps cancer stem cells to override apoptotic cell death through construction of blebbishields from apoptotic bodies and subsequent cellular transformation (sphere formation) from blebbishields through blebbishield-blebbishield fusion.^1–9^ Endocytosis drives the formation of serpentine filopodia, which help to position apoptotic bodies for membrane fusion.^5^ Inhibition of dynamin-dependent endocytosis using dynasore blocks blebbishield construction and promotes apoptotic body formation.^5^ These facts suggest the critical role of dynamin-dependent endocytosis in formation of serpentine filopodia and subsequent blebbishield formation from apoptotic bodies. Improved understanding of the endocytosis regulators acting during the blebbishield emergency program is necessary to shed light on human tumorigenesis and develop therapeutics to block blebbishield formation.

RalBP1 (also known as RLIP76) is an endocytosis mediator that binds to RalA to orchestrate human tumorigenesis.^10^ Upon induction of apoptosis, protein complexes containing RalBP1, RalGDS, and K-Ras dissociate from E-cadherin to initiate endocytosis by leaving the plasma membrane.^5^ This fact firmly links RalBP1 to endocytosis during the blebbishield emergency program.

Von Hippel–Lindau (VHL) is a prominent tumor suppressor and is expressed as p19-VHL and p30-VHL isoforms.^11^ VHL has also been shown to play an oncogenic role by regulating dynamin-dependent endocytosis through nm23^12–14^ and inhibiting apoptosis in response to multiple signals.^15,16^ VHL also regulates the expression of VEGF and VEGFR2 (drivers of the blebbishield emergency program^1,2,5^), through multiple mechanisms in renal cell carcinoma, for which VHL is an established cause.^17–19^ However, the reason why VHL plays oncogenic as well as tumor suppressor roles is poorly understood. Thus we examined the relationship between VHL and RalBP1 in the context of the blebbishield emergency program.

Here we demonstrate that dimers and trimers of p19-VHL isoform interacted with RalBP1 in an endocytosis-dependent manner to construct blebbishields from apoptotic bodies. Expression of RalBP1, p19-VHL, and high-molecular-weight (HMW) VHL (>tetramer) was required for blebbishield-mediated transformation, demonstrating that p19-VHL is an oncogenic isoform. In contrast, p30-VHL monomer and dimer (60 kDa) played a tumor suppressor role by inhibiting the blebbishield emergency program. Furthermore, VHL target genes (direct or indirect) that suppress oxidative stress were elevated during the blebbishield emergency program, indicating that management of oxidative stress during the blebbishield emergency program helps cancer stem cells to override apoptosis. Thus, distinct oligomeric states of p19-VHL and p30-VHL isoforms determine their oncogenic or tumor suppressor roles during the blebbishield emergency program.

## Results

### VHL (p19-VHL) but not RalBP1 is expressed as distinct oligomers in cancer cell lines

**Figure 1:**
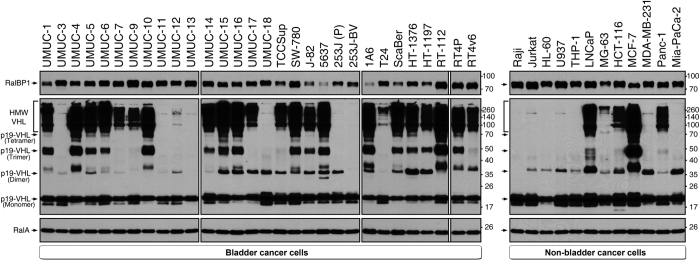
Expression analysis of RalBP1 and VHL in various human cancer cell lines. Expression of RalBP1 and p19-VHL in various human bladder cancer cell lines (left panels) and non-bladder cancer cell lines (right panels) by western blotting. RalA served as a loading control. All the blots were aligned on the basis of molecular weight. Note the oligomeric patterns of p19-VHL isoform (monomer, dimer, trimer, tetramer, and HMW VHL), RT4P, and RT4 bladder cancer cells. Suspension cells: Raji, Jurkat, HL-60, and U937. SDS-PAGE: 15% gel; 30 *μ*g per lane.

RalBP1 is tightly linked to suppression of apoptosis and promotion of tumorigenesis.^20,21^ Apoptotic cancer stem cells use RalBP1 in dynamin-dependent endocytosis to suppress apoptosis during the blebbishield emergency program.^5^ Although VHL is generally portrayed as a tumor suppressor,^11^ VHL is also known to play an oncogenic role by inhibiting apoptosis^15^ and to regulate dynamin-dependent endocytosis.^12^ Hence, we examined the expression of RalBP1 and VHL in 30 human bladder cancer cell lines and in 12 human non-bladder cancer cell lines from hematological and solid tumor types. We found that RalBP1 was ubiquitously expressed in all cell lines tested and that J-82, 5637, 1A6, and ScaBER cells expressed lower amounts of RalBP1 (Figure 1).

VHL on the other hand, expressed p19 isoform (19-kDa monomer) in all cell lines tested but exhibited cell line-dependent oligomeric states, including dimers (38 kDa), trimers (57 kDa), tetramers (76 kDa), and HMW VHL (95 kDa to 260 kDa) (Figure 1). In contrast, VHL p30 isoform was not expressed in any of the cell lines tested (in live cells, basal expression). Cells with epithelial phenotype expressed oligomeric VHL, especially HMW VHL, whereas cells with mesenchymal phenotype lacked expression of HMW VHL (UMUC-3 and 253J-BV cells) or had feeble expression of HMW VHL (T24 cells) (Figure 1). Similar to mesenchymal cells, hematopoietic cells (Raji, Jurkat, HL-60, U937, and THP-1) lacked HMW VHL expression, coinciding with their suspension growth in nature (Figure 1). Dimeric, trimeric, and tetrameric VHL showed variations in expression irrespective of epithelial or mesenchymal nature of the cells (Figure 1). Taken together, these results demonstrated that p19-VHL was expressed in all cancer cell lines tested, whereas none of the tested cell lines expressed p30-VHL, and that p19-VHL was expressed in different oligomeric states, including monomers, dimers, trimers, tetramers, and HMW VHL.

### Dimeric and trimeric p19-VHL interact with RalBP1 in an endocytosis-dependent manner specifically during blebbishield formation

**Figure 2:**
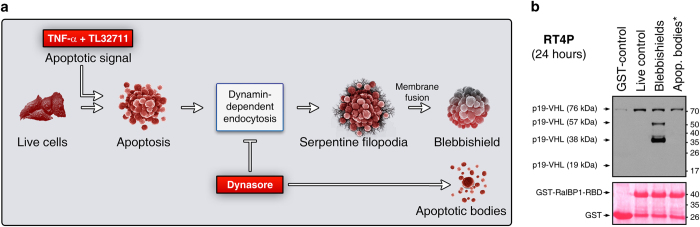
RalBP1 interacts with dimers and trimers of p19-VHL during blebbishield formation but not during apoptosis. (**a**) Schematic showing that inhibition of dynamin-dependent endocytosis can inhibit blebbishield formation and promote apoptotic body formation in RT4P cancer cells (please see text for reference). (**b**) GST-pulldown assay using GST or GST-RalBP1-RBD (RalA binding domain) as bait to show the interaction of p19-VHL oligomers with GST-RalBP1-RBD. Note: HMW VHL and monomeric p19-VHL did not interact with GST-RalBP1-RBD. The lysates were collected at 24 h, and the pulldown assay was done for 1 h. Apop. bodies*, apoptotic bodies (includes cell body too) generated using dynasore to inhibit blebbishield formation after apoptosis.

Both RalBP1^22^ and VHL^12^ are known to play roles in dynamin-dependent endocytosis. Hence, we hypothesized that RalBP1 and VHL interact to regulate endocytosis during the blebbishield emergency program. We previously demonstrated that incubation of RT4P cells (the cell line in which the blebbishield emergency program was discovered^1^) with a combination of TNF-*α* and Smac mimetic TL32711 induces apoptosis and endocytosis-dependent blebbishield formation, whereas inhibition of dynamin-dependent endocytosis with dynasore blocks blebbishield formation to promote apoptotic body formation^5^ (Figure 2a). We exploited this experimental setup to generate blebbishields (exhibits dynamin-dependent endocytosis) and apoptotic bodies (dynamin-dependent endocytosis inhibited with dynasore) at 24 h and examined the interaction of RalBP1 and VHL in RT4P cells using GST-RalBP1-RBD as bait. The results revealed that tetrameric p19-VHL (76 kDa) interacted with RalBP1-RBD in live cells, blebbishields, and apoptotic bodies (Figure 2b). Interestingly, dimeric (38 kDa) and trimeric (57 kDa) p19-VHL interacted with RalBP1-RBD only in blebbishields, and these interactions were inhibited by dynasore-mediated inhibition of dynamin-dependent endocytosis (i.e., in apoptotic bodies) (Figure 2b). Of note, neither monomeric p19-VHL nor HMW VHL interacted with RalBP1-RBD under any of the conditions tested. Taken together, these data confirmed that the interaction of p19-VHL dimers and trimers with RalBP1 occurred specifically during blebbishield formation.

### Tetrameric but not dimeric or trimeric p19-VHL interacts with RalBP1 in non-apoptotic and mitotic cells

**Figure 3:**
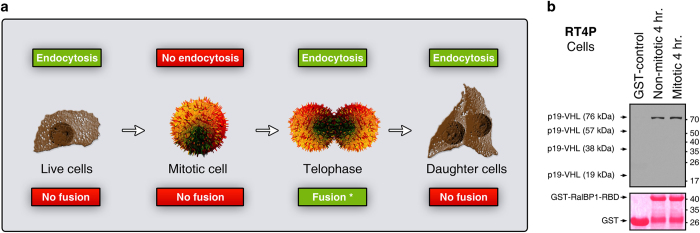
Tetrameric but not dimeric and trimeric VHL interacts with RalBP1 in non-apoptotic and mitotic cells. (**a**) Schematic showing the relationship of endocytosis to cell cycle phase. Endocytosis is inhibited during mitotic phase (metaphase) and can resume endocytosis during late mitosis such as during telophase. *Although telophase contributes to cleavage furrow formation, cells at this stage can undergo fusion. (**b**) GST-pulldown assay using GST or GST-RalBP1-RBD (RalA binding domain) as bait to show interaction of p19-VHL isoforms with GST-RalBP1-RBD in non-apoptotic and non-mitotic (G1 and S phases), and mitotic cells (M phase). Note: the lysates were collected at 24 h, and the pulldown assay was done for 1 h.

We next examined whether VHL interacts with RalBP1 in non-apoptotic and mitotic cells. For this purpose, we used non-apoptotic/non-mitotic live cells and freshly isolated mitotic cells (metaphase). Freshly isolated mitotic cells (metaphase) do not exhibit endocytosis (Figure 3a). Cells in the late stages of mitosis, such as telophase, exhibit endocytosis and fusion (Figure 3a), but we could not obtain cells in telophase because no agents were known to arrest cells at telophase without causing apoptosis.^23^ Hence, we tested only metaphase mitotic cells with live non-mitotic cells. We found that VHL tetramers but not dimers and trimers interacted with RalBP1-RBD in non-apoptotic and non-mitotic cells, and metaphase mitotic cells (Figure 3b). Again, neither HMW VHL nor monomeric VHL interacted with RalBP1-RBD. Together, these data further confirmed that the interaction between dimeric and trimeric VHL, and RalBP1 occurred specifically during blebbishield formation.

### Expression of p30-VHL oligomeric states and loss of expression of RalBP1 correlate with inhibition of transformation from blebbishields

**Figure 4:**
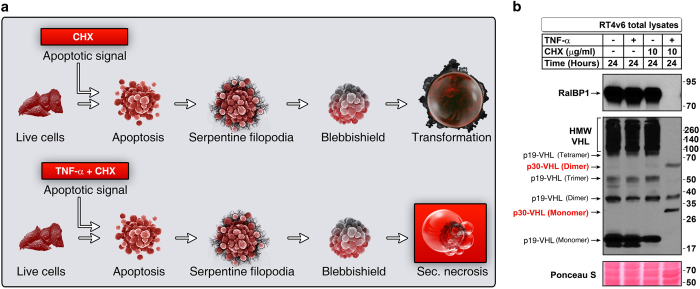
Expression of p30-VHL isoform and loss of expression of RalBP1 correlate with inhibition of transformation from blebbishields. (**a**) Schematic showing the difference in outcomes between CHX-induced apoptosis, which culminates in blebbishield formation and cellular transformation from blebbishields, and TNF-*α*+CHX-induced apoptosis, in which transformation is inhibited by secondary necrosis (see text for reference). (**b**) RT4v6 cells were treated with CHX or TNF-*α*+CHX for 24 h, and the expression of RalBP1 and VHL was analyzed by western blotting. Note the expression of p30-VHL monomer and dimer specifically in TNF-*α*+CHX-treated cells. Note: lanes 3 and 4 but not lanes 1 and 2 represent conditions after apoptosis.

We next investigated the status of expression of RalBP1 and VHL oligomers in the transformation phase of the blebbishield emergency program. For these experiments, we used RT4v6 cells, which are more efficient than RT4P cells in sphere formation from blebbishields.^1^ In RT4v6 cells, blebbishields generated by 10 *μ*g/ml cycloheximide (CHX) were able to form spheres (transformation from blebbishields), whereas blebbishields generated using a combination of TNF-*α* and CHX were not able to form spheres because of increased secondary necrosis induction in blebbishields^6^ (Figure 4a). We exploited that difference in sphere formation capacity to explore the expression of RalBP1 and VHL oligomers during transformation phase of blebbishield emergency program. The results revealed that the combination of TNF-*α* and CHX abrogated the expression of RalBP1 and generated a distinct set of VHL oligomers (p30-VHL and its 60-kDa dimer) (Figure 4b). Interestingly, HMW VHL, monomeric p19-VHL, and trimeric p19-VHL were lost in the blebbishields generated by the combination of TNF-*α* and CHX (Figure 4b). In contrast, the blebbishields generated by CHX expressed RalBP1 and all the oligomeric states of p19-VHL, but not p30-VHL or its 60-kDa dimer (Figure 4b). These data demonstrated that the expression of RalBP1, dimeric, and trimeric p19-VHL oligomeric states are pivotal for transformation from blebbishields and that p30-VHL may play a tumor suppressor role by inhibiting the blebbishield emergency program.

### Expression of RalBP1 and p19-VHL oligomeric states play an oncogenic role and expression of p30-VHL plays a tumor suppressor role during the blebbishield emergency program

**Figure 5:**
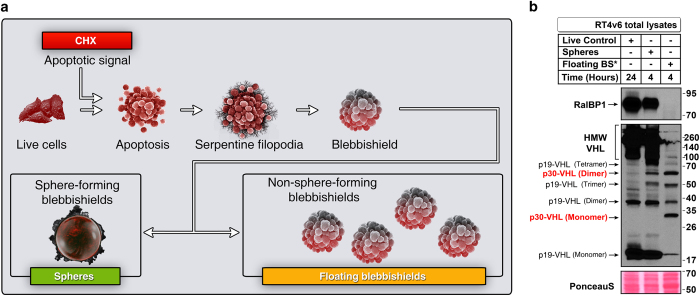
Expression of p19-VHL oligomeric states and expression of RalBP1 play an oncogenic role and expression of p30-VHL oligomeric states plays a tumor suppressor role during the blebbishield emergency program. (**a**) Schematic showing the method to isolate spheres (sphere-forming blebbishields) and non-sphere-forming blebbishields from RT4v6 cells (see text for reference). (**b**) RT4v6 cells were treated with CHX for 24 h to allow apoptosis induction and blebbishield formation, and blebbishields were isolated and plated for 4 h to allow sphere formation. The sphere-forming blebbishields and non-sphere-forming blebbishields were isolated and examined for the expression of RalBP1 and VHL by western blotting. Note the expression of p30-VHL monomer and dimer, and loss of expression of RalBP1, HMW VHL, and p19-VHL monomer specifically in non-sphere-forming blebbishields. Note: lanes 2 and 3 but not 1 represent conditions after apoptosis. Floating BS*, floating non-sphere-forming blebbishields.

Although loss of RalBP1 and monomeric, trimeric, and HMW p19-VHL correlated with loss of transformation from blebbishields generated by the combination of TNF-*α* and CHX (Figure 4b), there exists a possibility that these oligomers could have been lost as a result of excessive secondary necrosis. Thus, we used CHX (without TNF-*α*) to generate blebbishields for 24 h and then allowed the blebbishields for sphere formation for further 4 h. We then isolated spheres and non-sphere-forming blebbishields to study the expression of RalBP1 and VHL oligomeric states (Figure 5a). We chose 4 h for this experiment because longer duration of incubation induces secondary necrosis in non-sphere-forming blebbishields (Figure 5a). The results revealed that RalBP1 expression was completely abrogated in non-sphere-forming blebbishields but not in sphere-forming blebbishields (Figure 5b). Likewise, expression of HMW VHL and monomeric VHL was greatly reduced in non-sphere-forming blebbishields but not in sphere-forming blebbishields (Figure 5b). Furthermore, expression of p30-VHL monomer and dimer (60 kDa) was specifically increased in non-sphere-forming blebbishields compared to sphere-forming blebbishields (Figure 5b). Importantly, trimeric VHL was not lost in non-sphere-forming blebbishields (Figure 5b), suggesting that the loss of trimeric VHL observed in blebbishields generated by the combination of TNF-*α* and CHX (Figure 4b) is due to secondary necrosis. This interpretation also helps to confirm that loss of HMW VHL and monomeric VHL was not due to secondary necrosis because the trimeric VHL is not lost (Figure 5b). The complete loss of RalBP1 expression in non-sphere-forming blebbishields precluded evaluation of RalBP1 interaction with oligomeric states of VHL with RalBP1. Taken together, these results validated and confirmed that monomeric p19-VHL and HMW VHL play pivotal roles in the transformation phase of the blebbishield emergency program and that p30-VHL and its 60-kDa dimer inhibit the blebbishield emergency program.

### Evaluation of VHL genetic signature reveals that an oxidative stress suppressor network operates during the blebbishield emergency program

**Figure 6:**
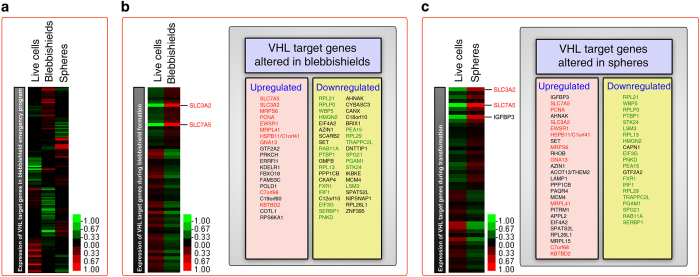
An oxidative stress suppressor network is provoked during the blebbishield emergency program. (**a**) Microarray gene expression heatmap of VHL target genes (may include direct and indirect targets) showing the subsets of VHL target genes specifically expressed in live RT4P cells, RT4P-blebbishields, and RT4P-transformed spheres. (**b**) Microarray gene expression heatmap of VHL target genes showing the genes altered in blebbishields compared to live cells. Genes listed in red were also upregulated in spheres. Genes listed in green were also downregulated in spheres. Genes listed in black were altered specifically during blebbishield formation phase. (**c**) Microarray gene expression heatmap of VHL target genes showing the genes altered in spheres compared to live cells. Genes listed in red were also upregulated in blebbishields. Genes listed in green were also downregulated in blebbishields. Genes listed in black were altered specifically during transformation from blebbishields. Please see Supplementary Table 1 for the complete list of gene alterations.

Although VHL is a known tumor suppressor,^11^ our findings suggest that VHL can have an oncogenic or tumor suppressor role depending on the expression of isoforms and their oligomeric state. VHL interacts with transcriptional elongation factors elongin-B and elongin-C^24,25,26^ to form a complex, and to act as a tumor suppressor.^27^ Since VHL-regulated genes (could be direct or indirect targets) were identified previously,^28^ we examined VHL-regulated gene expression (137 genes) in the blebbishield emergency program using our microarray transcriptome profiling data to get insight into the functional targets of VHL during blebbishield formation and transformation from blebbishields. Among the 137 VHL-regulated genes examined, live cells, blebbishields, and spheres that are transformed from blebbishields had expressed their own but less overlapping subsets of genes (Figure 6a; Supplementary Table 1). Blebbishields had 20 VHL target genes upregulated (expression differences high to low in the following order *SLC7A5, SLC3A2, MRPS6, PCNA, EWSR1, MRPL41, HSPB11/C1orf41, GNA13, GTF2A2, PRKCH, ERRFI1, KDELR1, FBXO18, FAM53C, POLD1, C7orf68, C19orf60, KBTBD2, COTL1,* and *RPS6KA1*) and 39 VHL target genes downregulated (expression differences high to low in the following order *RPL21, RPLP0, WBP5, HMGN2, EIF4A2, AZIN1, SCARB2, SET, RAB11A, PTBP1, GMFB, RPL13, PPP1CB, CKAP4, FXR1, IRF1, C12orf10, EIF3G, SERBP1, PNKD, AHNAK, CYBASC3, CANX, C18orf10, BRIX1, PEA15, RPL29, TRAPPC2L, DNTTIP1, SPG21, PGAM1, STK24, IKBKE, MCM4, LSM3, SPATS2L, NIPSNAP1, RPL26L1,* and *ZNF395*) compared to RT4P live cells (Figure 6b). Likewise, spheres transformed from blebbishields had 26 VHL target genes upregulated (expression differences high to low in the following order *IGFBP3, SLC7A5, PCNA, AHNAK, SLC3A2, EWSR1, HSPB11/C1orf41, SET, MRPS6, RHOB, GNA13, AZIN1, ACOT13/THEM2, LAMP1, PPP1CB, PAQR4, MCM4, MRPL41, PITRM1, APPL2, EIF4A2, SPATS2L, RPL26L1, MRPL15, C7orf68,* and *KBTBD2*) and 21 VHL target genes downregulated (expression differences high to low in the following order *RPL21, WBP5, RPLP0, PTBP1, STK24, LSM3, RPL13, HMGN2, CAPN1, EIF3G, PNKD, PEA15, GTF2A2, FXR1, IRF1, RPL29, TRAPPC2L, PGAM1, SPG21, RAB11A,* and *SERBP1*) compared to RT4P live cells (Figure 6c).

Interestingly, a subset of these genes remained upregulated or downregulated in both phases of the blebbishield emergency program (gene names in red (upregulated) and green (downregulated) in Figures 6b and c). We considered these genes to have an essential role in the blebbishield emergency program. Among the genes upregulated in both phases were *SLC3A2*, a protein product of which dictates transformation by modulating cell adhesion by interacting with *β*1 integrins;^29,30^ *SLC7A5*, a protein product of which is linked to transformation of leukemic cells;^31^ and *PCNA*, a protein product of which orchestrates oxidative DNA damage repair.^32,33^ Likewise a subset of genes was altered in either the blebbishield formation phase (e.g., *RPS6KA1*) or the transformation phase (e.g., *IGFBP3*) but not both (Figures 6b and c).

Importantly, *SLC3A2* (codes for the protein CD98hc),^34^ *SLC7A5* (codes for the protein l-type amino-acid transporter, a binding partner of SLC3A2^35,36^), and *IGFBP3*^37^ are linked to amino-acid transport, oxidative stress management, and tumorigenesis.^38^ Amino acids are essential for oxidative stress suppression/management,^39^ and thus amino-acid transporters such as SLC3A2, SLC7A5, and IGFBP3 might team up in oxidative stress management in apoptotic blebbishields. This concept is supported by findings from our previous studies, which confirmed the critical roles of reactive oxygen species (ROS),^3^ amino acids,^1^ and ROS management^4^ in the blebbishield emergency program. Taken together, these data revealed that an oxidative stress management network (*SLC3A2, SLC7A5,* and *IGFBP3*) under the control of p19-VHL performs an oncogenic role by overriding apoptosis in cancer stem cells.

## Discussion

VHL, a prominent tumor suppressor, often gets inactivated in renal cell carcinoma.^11^ VHL has two isoforms, p19-VHL and p30-VHL, generated by alternative initiation codons.^11,40–42^ We for the first time demonstrated an oncogenic role for p19-VHL isoform by showing its ability to override apoptosis through its oligomeric states and demonstrated a tumor suppressor role for p30-VHL by showing that its expression inhibits transformation phase of blebbishield emergency program. The interaction of dimeric and trimeric p19-VHL with the tumorigenic engine RalBP1 and the requirement of expression of HMW and monomeric p19-VHL for transformation from blebbishields adds more oncogenic flavor to the p19-VHL isoform. Of note, the blebbishield emergency program has been demonstrated to be a tumorigenic mechanism after apoptosis.^1^ RalBP1 has repeatedly been found to be required for human tumorigenesis.^10,20,43,44^ RalBP1 has also been found to co-ordinate endocytosis of K-Ras, PKC-*ζ*, and cdc42 to regulate formation of serpentine filopodia (which in turn construct blebbishields from apoptotic bodies) and internalize VEGFR2 by endocytosis to drive cellular transformation from blebbishields.^5^

The VHL oligomers are stable in response to heat (boiling samples before SDS-PAGE), SDS-mediated denaturation (during SDS-PAGE) and resistant to *β*-mercaptoethanol-mediated reduction (to relieve di-sulfide linkages within and between proteins). Thus the VHL oligomers are formed possibly by covalent protein modifications, but the oligomers are held together during SDS-PAGE, independent of di-sulfide linkage(s) between VHL proteins. SDS-resistant complexes are formed between proteins when ATP levels go down^45^ and K-Ras, BAD, p27, Bax, and Bak are known to oligomerize and boost glycolysis,^6^ which in turn can generate ATP. Oxidative stress activates glycolysis^46^ and glycolysis in turn promotes survival through blebbishield emergency program.^6^ VHL indeed is linked to ATP levels and glucose uptake^47^ raising a possibility that VHL oligomerization may be related to ATP/glycolysis. Although dynamin-dependent p19-VHL interaction with RalBP1 highlights the endocytic function of VHL to construct blebbishields, elucidating the exact cause of VHL oligomerization needs additional studies.

Dynamin-dependent endocytosis does occur in non-mitotic live cells; however, the dynamin-dependent endocytosis that occurs during blebbishield formation is distinct from the dynamin-dependent endocytosis that occurs in non-mitotic live cells because the interaction of dimeric and trimeric VHL is detected only in blebbishields. Furthermore, this interaction was inhibited by dynasore (not detected in apoptotic bodies), and hence this interaction is not for apoptotic process. Hence, the interaction of p19-VHL dimers and trimers with RalBP1-RBD is oncogenic and specific to the blebbishield formation phase of the blebbishield emergency program.

The loss of expression of RalBP1, monomeric p19-VHL, and HMW VHL in non-sphere-forming blebbishields underscores the pivotal role of these proteins in blebbishield-mediated cellular transformation. The appearance of p30-VHL and its 60-kDa dimer in non-sphere-forming blebbishields further underscores the inhibitory nature of p30-VHL over blebbishield-mediated cellular transformation.

Our identification of increase in *SLC3A2, SLC7A5, PCNA*, and *IGFBP3* mRNA expression in the blebbishield emergency program indicates that these VHL target genes are linked to amino-acid transport, DNA damage repair, oxidative stress suppression, and tumorigenesis^37^ by ultimately regulating cellular transformation after apoptosis. Our previous studies on ROS during the blebbishield emergency program support this concept.^3,4,48^ It should be noted that suppression of ROS could play a dual role depending on the stage at which ROS suppression is initiated: ROS suppression before apoptosis could inhibit apoptosis and thus inhibit the blebbishield emergency program, whereas ROS suppression during or after commencement of apoptosis could be oncogenic because it helps to reduce the damage induced by ROS and save the cells from death by promoting blebbishield-mediated cellular transformation.

**Figure 7:**
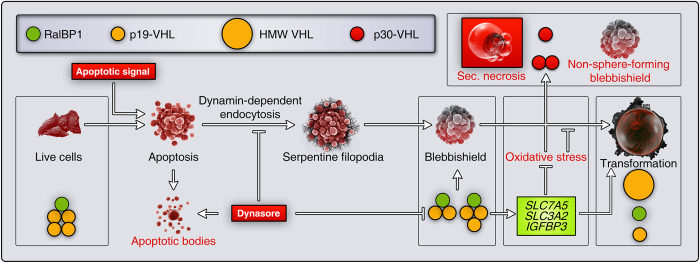
Expression and interactions of VHL isoforms and their oligomers during the blebbishield emergency program. Schematic showing the dynamic roles of p19-VHL, p30-VHL, and RalBP1 to regulate different phases of the blebbishield emergency program. Events labeled in red are fatal. RalBP1 interacts with p19-VHL dimers and trimers to regulate the blebbishield formation phase and can be inhibited by inhibition of dynamin-dependent endocytosis using dynasore. The VHL target genes that suppress ROS/oxidative stress are elevated in the blebbishield emergency program and hence contribute to transformation from blebbishields. Thus, these VHL target genes give VHL an oncogenic function. The p30-VHL monomer and dimer are associated with inhibition of transformation from blebbishields, and thus assume a tumor suppressor role.

In summary, we identified the reason why VHL behaves as both an oncogene and a tumor suppressor. Specifically, we identified roles of p19-VHL in transformation from blebbishields, p19-VHL dimer, and trimer in endocytosis-dependent RalBP1 interaction in blebbishields, p19-VHL tetramer in RalBP1 interaction in live cells, HMW VHL in transformation from blebbishields, p30-VHL in preventing cellular transformation from blebbishields, and 60-kDa dimer of p30-VHL in preventing cellular transformation from blebbishields (Figure 7). Thus, our findings provide deeper understanding of the roles of VHL isoforms and their distinct oligomeric states in cancer.

## Materials and methods

### Cells and their maintenance

RT4 cells (American Type Culture Collection; HTB-2, referred to as RT4 parental (RT4P) in this study), RT4v6 cells (RT4P cells enriched for cancer stem cells by serially passaging in mice six times^1^), fingerprint-verified bladder cancer cells (a gift from Dr. David J McConkey, The University of Texas MD Anderson Cancer Center), and non-bladder cancer cells (gifts from Drs Candelaria Gomez-Manzano, Felipe Samaniego, Isaiah J Fidler, Sun-Jin Kim, Guillermo Garcia-Manero, Xiongbin Lu, and Chun Li^1^) were cultured in MEM/RPMI with 10% fetal bovine serum, l-glutamine (MEM component), pyruvate, non-essential amino acids, vitamins, penicillin, and streptomycin supplements.

### Reagents

Nocodazole (M1404: used at 400 ng/ml) and dynasore (D7693: used at 100 *μ*M) were purchased from Sigma (St. Louis, MO, USA). Antibody to VHL (556743) was purchased from BD Biosciences (San Jose, CA, USA). Antibodies to RalA (4799) and RalBP1 (5739) were purchased from Cell Signaling Technology (Beverly, MA, USA). Recombinant human TNF-*α* (210-TA: used at 17 ng/ml) was purchased from R&D Systems (Minneapolis, MN, USA). Smac mimetic TL32711/birinapant (A-1901: used at 100 nM) (previously known as compound-C^49^) was purchased from Active Biochem (Maplewood, NJ, USA). CHX (239763: used at 10 *μ*g/ml) was purchased from Calbiochem (San Diego, CA, USA).

### Plasmids

Bacterial expression plasmid pGEX-GST-RBD (Ral binding domain of RalBP1; amino acids 403–499) was a gift from Dr. Michael White (UT Southwestern, Dallas, TX, USA)^50,51^ and Dr. Christopher Counter (Duke University School of Medicine, Durham, NC, USA).^10^ The pGEX-4T control plasmid was a gift from Dr. Santosh Chauhan (University of New Mexico, USA).

### Preparation of mitotic and non-mitotic cell extracts

RT4P cells (1×10^7^) were arrested at mitotic phase using 400 ng/ml nocodazole treatment for 4 h (longer incubations may induce apoptosis) in 15 ml of 15% FBS containing MEM, and the mitotic cells were then collected by the 'mitotic shake-off' method as described previously.^5^ Remaining cells, which included cells in all phases of the cell cycle except mitosis to telophase, were collected by scraping the cells in ice-cold serum-free MEM (to prevent serum-induced signaling). Both populations (mitotic and non-mitotic) were centrifuged at 1200 r.p.m. for 3 min, washed with PBS at 1200 r.p.m. for 3 min, and subjected to lysis, GST-pulldown, and western blotting as described below.

### RalBP1 interaction assays (GST-RalBP1-RBD pulldown assays)

DH5*α* strains were transformed with pGEX constructs; induced with 1 mM IPTG (I6758; Sigma) for 4 h at 37 °C; lysed using sonication (output: 8; 15- s bursts, twice) in bacteria lysis buffer (20 mM HEPES (pH 7.5), 120 mM NaCl, 10% (v/v) glycerol, 2 mM EDTA, 1 mM DTT, 0.5% NP-40, 1×-protease inhibitor cocktail, and 1 mM PMSF); clarified at 13 000 r.p.m. for 10 min; bound to glutathione sepharose-CL4B for 1 h at 4 °C; washed six times using bacteria lysis buffer; added to 200 *μ*g/200 *μ*l lysates prepared in whole-cell lysis buffer (50 mM Tris-HCl (pH 7.4), 150 mM NaCl, 5 mM EDTA, 25 mM NaF, 1% Triton-X 100, 1% NP-40, 0.1 mM Na3VO4, 12.5 mM *β*-glycerophosphate, 1 mM PMSF, and 1×-complete protease inhibitor cocktail) diluted with 200 *μ*l of magnesium-containing lysis buffer (25 mM HEPES (pH 7.5), 150 mM NaCl, 1% (v/v) NP-40, 0.25% (w/v) sodium deoxycholate, 10% glycerol, 20 mM MgCl_2_, 1 mM EDTA, 1×-protease inhibitor cocktail, and 1 mM PMSF); and incubated for 1 h at 4 °C. Pulldown complexes were washed four times with magnesium-containing lysis buffer before being subjected to SDS-PAGE and immunoblotting with VHL antibody. The protein interactions from this publication have been submitted to the IMEx consortium (http://www.imexconsortium.org) through IntAct^52^ and assigned the identifier IM-25635.

### Western blotting

Cells/blebbishields/spheres were lysed using whole-cell lysis buffer (50 mM Tris-HCl, pH 7.4; 150 mM NaCl; 5 mM EDTA; 25 mM NaF; 1% Triton-X 100; 1% NP-40; 0.1 mM Na3VO4; 12.5 mM *β*-glycerophosphate; 1 mM PMSF, and complete protease inhibitor cocktail (Roche, Clovis, CA, USA)) by incubation on ice for 30–40 min with intermittent vortexing every 10 min. The lysates were clarified at 13 000 r.p.m. for 10 min, and the supernatants were quantified using BCA assay (Pierce Biotechnology, Rockford, IL, USA) and subjected to SDS-PAGE and western blotting on nitrocellulose membranes before the membranes were blocked with 10% non-fat milk in PBS-Tween 20 (0.1%) and probed with antibodies in 10% milk in PBS-Tween 20. The 5× SDS-PAGE sample buffer used was as follows: 375 mM Tris, pH 6.8; 0.01% bromophenol blue; 10% glycerol; 2% SDS; and 12.5% *β*-mercaptoethanol.

### Evaluation of the endocytic requirement for VHL–RalBP1 interaction

RT4P cells were induced to undergo apoptosis and blebbishield formation using 17 ng/ml TNF-*α* plus 100 nM Smac mimetic TL32711 for 24 h in parallel to generation of apoptotic bodies (inhibition of blebbishield formation) using 17 ng/ml TNF-*α* plus 100 nM Smac mimetic TL32711 plus 100 *μ*M dynamin inhibitor dynasore for 24 h. Live cells, blebbishields, and apoptotic bodies were lysed, clarified, protein quantified (as described in the western blotting section above), and subjected to GST-RalBP1-RBD pulldown and western blotting (as described above). A dynasore-sensitive interaction in blebbishields was considered as an interaction driving blebbishield formation through dynamin-dependent endocytosis.

### Isolation of sphere-forming and non-sphere-forming blebbishields

Sphere-forming and non-sphere-forming blebbishields were isolated from RT4v6 cells as described previously.^3^ Briefly, cells were plated at a density of 200 000 cells/ml (13 ml/T-75 flask×four flasks), and at 24 h treated with CHX 10 *μ*g/ml for 24 h. The floating pyknotic blebbishield populations were then gently collected, pelleted down at 1200 r.p.m. for 3 min at room temperature, and re-plated in 150-mm plates with complete MEM for a further 4 h. The floating cells (non-sphere-forming blebbishields) were washed with complete MEM and collected separately, and the attached spheres (sphere-forming blebbishields) were scraped in fresh ice-cold complete MEM. Both types of blebbishields were pelleted at 3500 r.p.m. and washed with ice-cold PBS before lysis and western blotting.

### Microarray analysis

RNAs from RT4P cells, RT4P-blebbishields (apoptotic), and blebbishield-mediated transformed spheres (at 4 h: post-apoptotic but undergone fusion and attachment to substratum) were isolated using a mirVana kit (Ambion Austin, TX, USA) and subjected to whole-transcriptome microarray analysis (Illumina, San Diego, CA, USA, Chip version 4, catalog # BD-103-0204); data were quantile-normalized. The 137 VHL target gene list^28^ (Molecular Signatures Database:^53^ C2 curated gene sets, Jiang_VHL_Targets: Systematic name: M18850) was picked from our transcriptome data, log-transformed, median-centered genes, and genes clustered using average-linkage in Cluster v3.0, and represented as a heatmap using Java TreeView, to generate the blebbishield emergency program phase-specific expression of VHL target genes. The data set with probe ID is available as Supplementary Table 1
