# Supplementary material for: RalBP1 and p19-VHL play an oncogenic role, and p30-VHL plays a tumor suppressor role during the blebbishield emergency program
Source: Cell Death Discov. 2017 May 29;3:17023–. doi: 10.1038/cddiscovery.2017.23 (PMC5447132; doi:10.1038/cddiscovery.2017.23)
Supplement: Supplementary Information [file cddiscovery201723-s1.pdf]

Supplementary Table-1: Expression of VHL target genes in blebbishield emergency program				
**VHL target genes**	**Illumina ProbeID**	**RT4P-control**	**RT4P-Blebbishields**	**RT4P-Spheres**
ABCB8	3990592	99.5	103	94.8
ACOT13/THEM2	1580427	883.2	852.5	1120.6
AHDC1	4250376	94.9	97.9	108.4
AHNAK	450553	1752.8	1532.5	2977.7
AIMP1/SCYE1	3710632	94.9	87.6	92.1
ALPL	6100356	94.7	105.8	93
ANKH	2680253	112.1	96.6	101.5
APPL2	4180100	464.2	466.1	632.5
ARFGEF2	3610554	249.4	270.1	248.9
ARTN	6560494	94.8	124.7	122.9
AZIN1	2480039	1494.2	883.9	1819.7
BCL7B	6370593	397.8	457	396.1
BRIX1	2120445	2154.3	1978.8	2139.7
C12orf10	1260022	1139.1	861.8	1046.1
C18orf10	2690541	566.7	391	607.9
C19orf60	6760414	786.1	936.5	733.6
C1D	2480674	94.8	91.8	98.8
C1orf159	5570523	108.7	116.2	132.2
C21orf59	2320324	193.6	183.2	211.5
C3orf75	4210102	554.1	554.6	483.2
C6orf106	5900504	140.5	125.6	129.7
C7orf68	7320441	859.7	1010.3	961.7
CANX	5860477	445	267.8	347.9
CAPN1	1570672	1542.5	1615.4	1109.2
CDK2	4590064	1365.5	1454.6	1293.3
CERS5/LASS5	6330356	249.6	225.8	266.6
CKAP4	4490528	992.5	666.7	914.1
CMTM3	4880184	120.2	133.4	126.6
COTL1	1070678	353.1	446.3	395.1
CYBASC3	6940152	515.6	308.4	570.7
DGCR6L	5420014	439.7	437.3	466.3
DNAJC1	2450678	124.4	101.7	104.1
DNTTIP1	2100678	698.2	560.6	700.3
EBPL	5490477	411.6	451.5	404
EIF3G	2190377	1407.8	1150.2	1057.8
EIF4A2	4640689	4900.2	3895.5	5060.3
ELANE	7650497	88.2	96.1	100.3
ENO2	50402	170.4	233.1	240.9
ERRFI1	7100639	518.1	787.5	427.8
EWSR1	670072	2037.4	2764.1	2840.3
FAM53C	2650019	719.3	926.4	725.3
FBXL5	110500	90.3	95.8	86.5
FBXO18	2470259	567	801.2	554.9
FXR1	520356	881.6	565.9	627.1
GATAD2B	2370576	126.9	118.2	124.2
GM2A	4280273	203.6	167.2	178.4
GMFB	5260044	1700.6	1249.6	1612.1
GNA13	3800730	970.5	1315.2	1310.8
GTF2A2	2450368	3484.9	3817.5	3192
HDAC6	130370	166.1	210.2	177.1
HIRIP3	1770717	269	293.9	202.8
HMGN2	4050541	3755	2490.6	3297.9
HN1L	430437	178.5	185.4	161

HSPB11/C1orf41	6450273	1069.4	1434.2	1487.7
IGFBP3	6590132	2813	2776.5	8880.6
IKBKE	2450762	237.7	118.2	172.4
INF2	2140360	189.3	213.8	133.7
IRF1	6250064	818.8	523	569.6
IRF2BPL/C14orf4	5290626	1074.5	1114.9	1149.2
KBTBD2	4920372	567.3	697	667.4
KDELR1	2510209	2716.8	2973.2	2667
KIAA1429	4560577	222.4	302.9	283.5
LAMP1	6270100	3019.5	2937.3	3236.9
LRCH1	940397	93.7	96.6	94
LRP1	2710286	91.4	92.8	100.6
LSM3	7380554	3177.2	3065.9	2614
MCM4	6020170	1202	1087.9	1385
MKI67	20364	134.2	105.9	122.6
MPHOSPH6	2260703	312.9	354.5	374.2
MRPL15	4760520	878.8	955.5	990.3
MRPL41	4780040	1334.7	1939.4	1514.2
MRPL53	2750309	553.2	523.1	482.5
MRPS36	3420079	183.6	148.9	152
MRPS6	6400195	2750	3544.9	3130.3
MT1L	5910477	102.5	105.5	103.6
MTHFD1	1660270	283.9	249.3	275.9
NFE2L1	4210176	378.7	420.1	471.3
NIPSNAP1	5490131	609.8	500.6	536.8
NPM1	6020192	97.6	95	94.4
OXA1L	6900402	1056.6	1017.9	1039.6
PAQR3	6960019	345.4	421.3	366.5
PAQR4	3460132	309.9	297.8	496.4
PATZ1	2320521	118.2	99.2	142.7
PCNA	6900079	1963.7	2715.4	3277.4
PDGFRA	3130240	94.4	94	97.1
PEA15	3610228	1153	992.8	824.6
PECR	6200255	115.4	123.1	181.6
PES1	1500066	250	285.4	314.5
PGAM1	3940592	1947.7	1823.6	1804.9
PHF17	6220411	216.1	222.9	249.9
PITRM1	1510072	353.3	311	525.7
PNKD	4730349	663.5	433.6	327.8
POLD1	290731	292.4	469.6	296.1
PPP1CB	6200369	784.2	434.5	986.4
PPP2R5D	5090035	301.4	328.2	322.1
PRKCH	3290731	724.3	1024.9	651.7
PRKRIP1	1710746	250.1	221	222.4
PTBP1	4260754	4076.3	3556.3	3126.9
RAB11A	780358	2111.4	1563.1	2004.3
RAB14	6250133	97.5	100.2	102.8
RANBP9	4610349	124.3	104.3	102.3
RHOB	3400332	498.1	470.4	854
RNF130	1510722	200.6	118.4	120
RPL13	1940743	944.8	518.7	400.6
RPL21	4920070	9502.7	6287.4	6557.9
RPL26L1	6130390	1246.7	1142.7	1362.3
RPL29	2350465	804.7	647	613.1
RPLP0	3940370	17842.5	15065.1	16853.8

RPS6KA1	4730739	205.5	295.4	232.2
SCARB2	4810435	2066.5	1483.2	2018.4
SERBP1	4210754	644	395.5	546.8
SET	4230224	2913	2342.5	3328.2
SGSM3	270475	151.6	172.8	148.2
SIRT6	1850739	105.5	101.5	100.3
SLC39A13	1190706	101.4	103.3	90.9
SLC3A2	5420575	600.6	2207.5	1476.9
SLC7A5	270152	265.7	1893.1	2177
SPATS2L	7380221	1203.7	1093.9	1360.9
SPG21	7200403	766.9	642.4	648.9
SS18	6380465	607.7	575.5	632.2
STK24	6180050	3747.8	3628.1	2855.3
SYDE1	2370348	115.7	123.6	109.3
TBC1D13	730465	147.4	170.7	182
TCTN3	5910615	221.9	172.7	218.5
TES	2120402	142.1	165.7	144.2
TMEFF1	3120403	101	104.2	107.5
TMEM222	6980039	117.3	148.5	146.1
TMEM50A	540193	135.2	113.1	129.7
TNFSF10	870202	213.1	159.2	222.9
TRAPPC2L	1450082	1546	1399.8	1387.4
TRIP10	430431	165.2	176	147.6
TROAP	4760646	263.4	327.4	196.1
TSTA3	2350661	334.1	282.3	373.7
UACA	1980630	116.6	98	94.7
VAPB	990202	107.1	98.7	117.3
WBP5	7510731	3735.4	1895.5	1677.4
ZNF395	1980403	696.2	594.2	692.4
